# The Emerging Roles of ATP-Dependent Chromatin Remodeling Complexes in Pancreatic Cancer

**DOI:** 10.3390/cancers11121859

**Published:** 2019-11-25

**Authors:** Nesrin Hasan, Nita Ahuja

**Affiliations:** Department of Surgery, Yale University School of Medicine, New Haven, CT 06520, USA; nesrin.hasan@yale.edu

**Keywords:** pancreatic cancer, PDAC, pancreas, epigenetics, chromatin, chromatin remodeling, ATP-dependent chromatin remodeling complexes, SWI/SNF, ISWI, CHD, INO80

## Abstract

Pancreatic cancer is an aggressive cancer with low survival rates. Genetic and epigenetic dysregulation has been associated with the initiation and progression of pancreatic tumors. Multiple studies have pointed to the involvement of aberrant chromatin modifications in driving tumor behavior. ATP-dependent chromatin remodeling complexes regulate chromatin structure and have critical roles in stem cell maintenance, development, and cancer. Frequent mutations and chromosomal aberrations in the genes associated with subunits of the ATP-dependent chromatin remodeling complexes have been detected in different cancer types. In this review, we summarize the current literature on the genomic alterations and mechanistic studies of the ATP-dependent chromatin remodeling complexes in pancreatic cancer. Our review is focused on the four main subfamilies: SWItch/sucrose non-fermentable (SWI/SNF), imitation SWI (ISWI), chromodomain-helicase DNA-binding protein (CHD), and INOsitol-requiring mutant 80 (INO80). Finally, we discuss potential novel treatment options that use small molecules to target these complexes.

## 1. Introduction

Pancreatic cancer is an aggressive cancer with <10% survival at five years that is poised to become the second cause of cancer-related deaths by 2030 [1]. Currently, surgical resection is the only curative option, however >80% of the patients present with an unresectable tumor [2]. Absence of early diagnostic tools, chemoresistance, and lack of novel therapies contribute to the low survival rate. Although multiple studies have been done to characterize the disease, effective therapies that improve patient survival rate have not yet been developed. 

**Figure 1:**
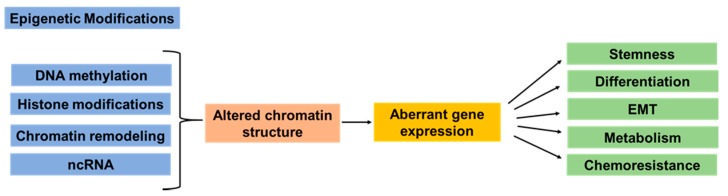
Simplified overview of the epigenetic modifications involved in cancer initiation, progression, and metastasis. Epigenetic modifications include DNA methylation, histone modifications, chromatin remodeling, and noncoding RNA (ncRNA)s. Multiple studies have demonstrated that epigenetic dysregulation in cancer has been linked to altered chromatin structure and modulation of accessibility of transcription factors to the DNA. These alterations have been associated with aberrant expression of genes related to cancer cell stemness, cell differentiation, epithelial–mesenchymal transition (EMT), cell metabolism, and response to therapeutic drugs.

Complex modifications are involved in pancreatic cancer initiation and progression. In addition to the mutations in the main oncogenes and tumor suppressors, the influence of the epigenetic dysregulation has been identified and is now increasingly being studied. Multiple studies highlighted the involvement of epigenetic dysregulation in cancer development, progression, and chemoresistance [3,4,5,6,7,8,9]. Epigenetics are changes that result in changes of gene expression without altering the DNA sequence and involve nucleosome remodeling, histone modifications, DNA methylation, and regulation through long noncoding RNAs (Figure 1). In eukaryotes, ~146 base pairs of genomic DNA is packed with an octamer of histone proteins to form the nucleosome, the basic unit of the chromatin [10]. Nucleosomes, linker histone protein, and non-histone proteins are further assembled into a highly organized chromatin structure that restricts access to the DNA [11]. Chromatin remodeling alters the chromatin structure and regulates accessibility of transcription factors and transcription machinery to the DNA, thus leading to dynamic regulation of gene expression. 

The two major enzyme groups involved in chromatin remodeling are ATP-dependent chromatin remodeling complexes that mobilize nucleosomes and histone-modifying enzymes that modify histones [12]. Histone-modifying enzymes covalently modify the N-terminal tails of the histones by using various post-translational modifications and alter the nucleosome structure and DNA-histone interactions [12,13,14]. Histone modifications are altered in cancer and contribute to cancer progression and metastasis [14,15]. Genome sequencing studies also reveal that chromatin regulatory proteins are highly mutated in cancer [16,17,18,19]. Specifically, in pancreatic cancer, chromosomal aberrations and/or mutations associated with ATP-dependent chromatin remodeling complexes have been detected in approximately one third of the samples [20,21], highlighting the involvement of aberrant chromatin remodeling in tumorigenesis. However, detailed reviews on the roles of the main subfamilies of the ATP-dependent chromatin-remodeling complexes in pancreatic cancer are limited. The scope of this review is to summarize the recent discoveries regarding the chromosomal alterations and mutations associated with subunits of the ATP-dependent chromatin remodeling complexes in pancreatic cancer and discuss their mechanistic roles and their targeting as a potential treatment strategy. 

## 2. Epigenetic Dysregulation in Pancreatic Cancer Development and Heterogeneity

The majority (>90%) of the diagnosed pancreatic cancer cases are pancreatic ductal adenocarcinoma (PDAC), which develops from the exocrine ductal cells and is associated with mutations in several driver oncogenes and tumor-suppressor genes. The tumor-initiating oncogenic KRAS mutations, which are found in >90% of PDAC cases, initiate the process for noninvasive precursor lesions. Mutations in tumor-suppressor genes SMAD4, TP53, and p16/CDKN2A are detected in 50–70% of the PDAC cases [21,22]. In addition to the sporadic PDAC described above, it is estimated that 5–10% of pancreatic cancers occur due to inherited germline mutations [23], the most prominent ones being in the BRCA2 and CDKN2A genes. 

PDAC development is associated with precursor lesions and the two major pathways that lead to PDAC include pancreatic intraepithelial neoplasms (PanIN) and intraductal papillary mucinous neoplasms (IPMN). Both pathways have distinct histological, genetic, and epigenetic changes associated with the multistep progression from low-grade precursor lesions to high-grade precursor lesions and invasive cancer [24,25]. The majority of the PDACs arise from PanINs, which are noninvasive microscopic flat or papillary intraepithelial lesions in the small intralobular pancreatic ducts [24]. In contrast, IPMNs are macroscopic cystic lesions that occur within the larger pancreatic ducts. IPMNs are heterogeneous and can be classified based on the site of origin and histological analysis. Some of the genetic changes in IPMNs are similar to the ones observed in PanINs and PDACs (KRAS, SMAD4, TP53, and p16/CDKN2A), whereas other mutations, such as activating GNAS and inactivating RNF43 mutations, are frequently observed only in IPMNs [26]. 

Multiple reports have highlighted that PDAC is associated with heterogeneity at both the genetic and epigenetic level, which might influence tumor progression. Several studies have classified the PDAC tumors based on transcriptional and genetic profiling [21,22,27,28,29,30,31]. The most widely used classification is by Moffitt et al., that defined two main tumor subtypes that are clinically and histopathologically different: the classical subtype and the basal subtype that is more aggressive with poorly differentiated tumors and poor outcome [29]. Multifactorial analysis and comparison of chromatin states and gene expression demonstrate that the two PDAC subtypes are associated with distinct chromatin states [32]. Another study by Hayashi and colleagues revealed that the basal subtype is associated with genetic alterations in the chromatin modifying genes, suggesting involvement of these genes in modulating tumor behavior [33]. Other studies have also highlighted that subtype development in PDAC is epigenetically driven and distinct epigenetic landscapes contribute to the PDAC heterogeneity [32,33,34,35]. Another interesting study compared the gene expression and DNA methylation by using PDAC patient-derived xenografts (PDXs) and demonstrated that the transcriptome and methylome have common patterns, highlighting that the main phenotypes in PDAC are established epigenetically [34]. Aberrant patterns of DNA methylation that can silence gene expression are commonly observed in PDAC, and they target tumor-suppressor genes involved in proliferation, apoptosis, cell adhesion, and major signaling pathways [7,31,32,36,37]. Supporting the fundamental role of epigenetic involvement in PDAC, another study concluded that epigenetic reprogramming involving DNA methylation and altered histone codes was associated with malignant gene expression and metastasis [35]. The role of epigenetic alterations in metastatic tumor progression was also confirmed by using PDAC mice models [38]. In addition, multiple sequencing studies have revealed chromosomal alterations and somatic non-silent mutations in components of the chromatin remodeling complexes in PDAC and other cancers [16,17,19,27,39,40,41,42]. Collectively, these reports suggest that epigenetic dysregulation and altered chromatin dynamics play an important role in PDAC. Comprehensive reviews of the roles of the four subfamilies of chromatin remodeling complexes in PDAC are lacking, presenting a knowledge gap, with the need for future studies. 

## 3. ATP-Dependent Chromatin Remodeling Complexes

ATP-dependent chromatin remodeling complexes have essential functions during development; therefore, it is not surprising that genomic aberrations in genes encoding chromatin remodeling components contribute to different malignancies, such as cancer, including PDAC [6,32,33,35,43,44,45]. Epigenetic reprogramming has significant roles in lineage specification during pancreas development, and the development-specific subunit expression is important for altering the functional activity of the complexes [46,47,48,49]. The majority of the studies have focused on epigenetic regulation in endocrine β-cells [46,48], and only a few reports have analyzed the role of SWI/SNF complexes in acinar and ductal cells [48,50]. Understanding the function of these complexes in pancreas development can also aid in identification of pathways that can be targeted in PDAC. 

**Figure 2:**
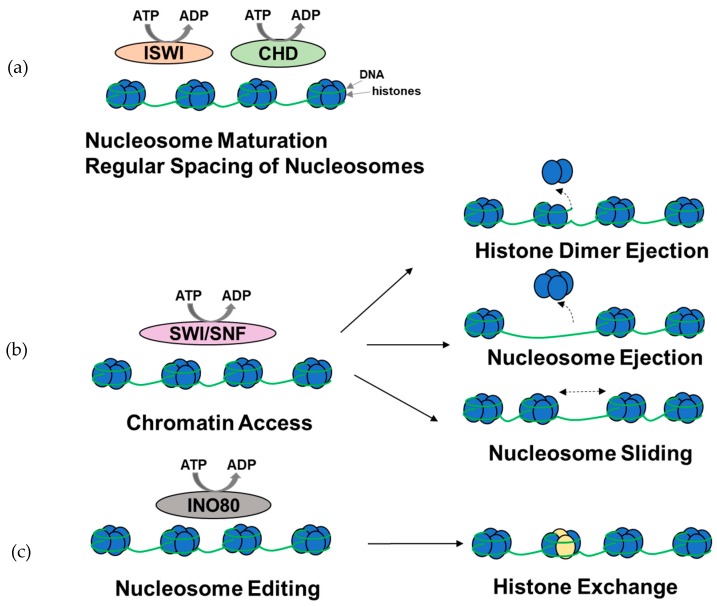
Overview of the functions of ATP-dependent chromatin remodeling complexes. (**a**) A subset of ISWI and CHD complexes are involved in nucleosome assembly, maturation, and spacing. (**b**) SWI/SNF complexes are primarily involved in histone dimer ejection, nucleosome ejection, and nucleosome repositioning through sliding, thus modulating chromatin access. (**c**) INO80 complexes are involved in histone exchange. It should be noted that the complexes might be involved in other chromatin remodeling functions (figure adapted from [52]).

ATP-dependent chromatin remodeling complexes bind to nucleosome cores and the surrounding DNA, and, using energy from ATP hydrolysis, they disrupt the DNA-histone interactions, slide or eject nucleosomes, alter nucleosome structures, and modulate the access of transcription factors to the DNA (Figure 2). In addition to modulating gene expression, some of the complexes are involved in nucleosome assembly and organization, following transcription at locations in which nucleosomes have been ejected, packing of DNA, following replication and DNA repair [45,51,52,53,54]. Based on the sequence homology of the catalytic ATPase and the accessory subunits, chromatin remodeling complexes are divided into four main subfamilies: SWItch/sucrose non-fermentable (SWI/SNF), imitation SWI (ISWI), chromodomain-helicase DNA-binding protein (CHD), and INOsitol-requiring mutant 80 (INO80) [52]. All of these complexes share a catalytic subunit containing a SWI2/SNF2-family ATPase domain that performs DNA translocation along the histone core of the nucleosome [52] and accessory subunits involved in target recognition, specificity, and modulation of the ATPase activity. 

The differences in subunit compositions of each complex are associated with the cell-type, tissue-specific, or development-related roles of each complex [47,52]. SWI/SNF and INO80 subfamily complexes form large protein assemblies comprising up to 15 subunits, whereas most ISWI complexes and a subset of the CHD complexes are formed with <4 subunits. 

### 3.1. SWI/SNF Subfamily

**Figure 3:**
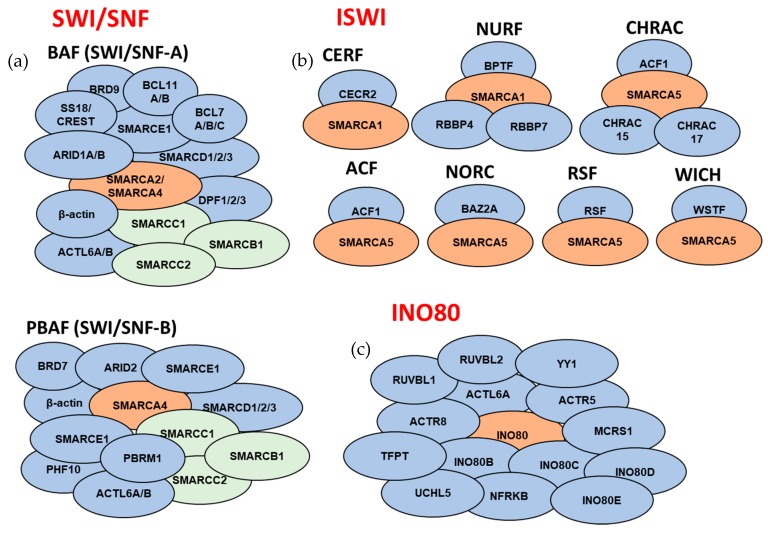
Overview of the subunit compositions of the ATP-dependent chromatin remodeling complexes. Subunits that comprise the mammalian (**a**) SWI/SNF complexes, (**b**) ISWI complexes, and (**c**) INO80 complex (for SWI-SNF complexes: orange color corresponds to catalytic ATPase subunits, green color corresponds to core subunits, and blue color corresponds to accessory subunits; for ISWI and INO80 complexes: orange color corresponds to catalytic ATPase subunits). For subunits that are separated by dashes, only one of the subunits is present in a given complex. Subunit composition might be different based on tissue/cell types. SWI/SNF noncanonical complex ncBAF and INO80 subfamily complexes p400 and SRCAP are not included in the schematic.

The SWI/SNF subfamily is involved in mobilizing the nucleosomes through repositioning, sliding, or ejection, and, typically, they facilitate chromatin access for transcription factors. The two main complexes are BAF and PBAF (Figure 3). Recently, a novel noncanonical complex, ncBAF, was identified [55]. The SWI/SNF complex is a multisubunit complex that includes a DNA-binding subunit (ARID1A, ARID1B, or PBRM1), an enzymatic ATPase subunit (BRM/SMARCA2 or BRG1/SMARCA4), three core subunits (SMARCB1, SMARCC1, and SMARCC2), accessory subunits, and BRM- or BRG1-associated factors (BAFs) that are essential for binding to DNA or proteins. The heterogeneity of the SWI/SNF complexes is associated with development and tissue-specific subtypes [44,56,57]. Multiple sequencing studies have identified the SWI/SNF complex as a major tumor suppressor in PDAC. Deletions or deleterious mutations in subunits of the SWI/SNF complexes were associated with 33–42% of the PDAC cases [20,21]. Genomic alterations were detected in multiple subunits of SWI/SNF complexes at varying frequencies [20,21,27] (Table 1). Additionally, SWI/SNF aberrations also modulate responsiveness to platinum-based treatment [58], indicating that detailed characterization of the human PDAC tumors can be used to identify biomarkers for improved treatment regimens. 

### 3.2. ISWI Subfamily 

ISWI complexes are involved in nucleosome organization following DNA replication and transcription. Specifically, they are involved in the maturation of DNA-histone complexes to nucleosomes, nucleosome sliding, and regular spacing of the nucleosomes [52]. Seven different mammalian ISWI complexes have been described so far: WICH, NoRC, RSF, ACF, CHRAC, NURF, and CERF (Figure 3). Each contains one of the two conserved ATPase subunits (SMARCA5 or SMARCA1) and accessory subunit(s) [53]. Different combinations of ATPases and accessory subunits might influence the chromatin remodeling reaction, such as the nucleosome spacing, and target the ISWI complex to different gene sets [53,59]. Most ISWI subfamily complexes are involved in repressing chromatin accessibility, whereas a subset such as the nucleosome remodeling factor (NURF) is involved in chromatin access and gene activation [53,60]. In addition, the ISWI complexes are involved in DNA damage response (DDR), which makes them a potential target in cancer [53]. 

### 3.3. CHD Subfamily

CHD subfamily comprises several complexes that have diverse functions, such as spacing of the nucleosomes, exposing the promoters, and editing the nucleosomes [52,61]. Most CHD members form multisubunit complexes and are involved in chromatin remodeling [61]. CHD3, CHD4, and CHD5 are components of the nucleosome remodeling deacetylase (NuRD) complex that is a transcriptional repressor and is the best characterized member of this subfamily. Multiple studies have investigated the role of NuRD in cancer [45,62,63,64]. The NurD complex and DNA methylation work cooperatively, demonstrating that both repressive histone marks and DNA hypermethylation are involved in the transcriptional silencing of tumor-suppressor genes [63,64], highlighting that multiple levels of epigenetic regulation are involved in cancer. 

### 3.4. INO80 Subfamily

INO80 multisubunit complexes have diverse functions that include transcriptional regulation, DNA replication, and DNA repair. They are involved in shifting nucleosomes and histone dimer or histone variant exchange. The INO80 subfamily includes the INO80, p400, and SRCAP complexes. INO80 subfamily complexes form large multisubunit complexes that include a catalytic ATPase (INO80, p400, or SRCAP), helicases (RUVBL1, RUVBL2), actin related proteins (ACTL6A, ACTR5, and ACTR8), and other subunits [51] (Figure 3). INO80 has roles in development, but its role in pancreas development is unclear [65,66]. Although the alteration frequency of INO80 subunits is high in multiple cancers, including PDAC [67] (Table 1), only a few studies have been done. The findings pointed to the tumor-promoting role of the INO80 complex in several cancers [67,68,69]. It has been associated with opening the chromatin state in cancer cells and in embryonic stem cells, and enhancer- or superenhancer-mediated oncogenic transcription [67,68,70]. In addition, a high co-occurrence of alterations in subunits of INO80 and mTORC1 was observed in PDAC and other cancers, suggesting that disruption of these pathways might contribute to the metabolic dysregulation involved in tumorigenesis [71]. 

## 4. Mechanistic Studies of the ATP-Dependent Chromatin Remodeling Complexes in PDAC 

Inactivating mutations in the SWI/SNF complexes are associated with various cancers, suggesting that they act as tumor repressors [16,72]. With slight exceptions, the involvement of the other three subfamilies in cancer has not been well characterized. Each complex is composed of multiple subunits that are associated with chromosomal alterations and/or mutations in PDAC (Table 1). The majority of the studies in PDAC have been focused on the role of few subunits of the SWI/SNF complex, whereas detailed mechanistic studies of the roles of the other subunits in PDAC are limited or missing. We summarize the current knowledge on the subunits of the ATP-dependent chromatin remodeling complexes in PDAC, and for cases that lack detailed mechanistic studies in PDAC, we include data from other cancers and/or stem cell studies, to provide evidence for their role. 

### 4.1. SWI/SNF Subfamily

#### 4.1.1. ARID1A 

ARID1A encodes a DNA-binding subunit of the human SWI/SNF complex and is the most frequently mutated subunit of the SWI/SNF complex in PDAC [49] (Table 1). ARID1A expression is decreased in PDAC (Table 2) and is associated with survival outcomes [21]. Studies in mice and cell lines have demonstrated that ARID1A is a tumor suppressor that represses KRAS-induced precancerous lesion formation and suppresses ductal proliferation [49,82]. Pancreas specific *Arid1a* deletion in mice induced inflammation, formation of PanINs, and mucinous cysts [49]. *ARID1A* deletion in vitro resulted in global increase of active histone marks and increase in protein expression through induction of Myc, as well as acinar, to ductal metaplasia [49]. Similarly, *Arid1a* deletion in mice PDAC tumors (mutant *Kras* and hemizygous *p53*) led to decreased cancer-specific survival and poorly differentiated tumors [49]. Further characterization of the derivative *Arid1a*-deleted cells revealed a stem-cell-like and EMT profile resulting in a migratory and mesenchymal phenotype [49]. Furthermore, *Arid1a* deletion in mice with pancreatic expression of activated KRAS resulted in IPMN that progressed to PDAC [49,82]. Mechanistically, *Arid1a* deletion inhibited the mTOR pathway, suppressed SOX9 expression, and led to dedifferentiation of pancreatic ductal cells [82]. 

Another interesting study demonstrated that postnatal acute silencing of *Arida1a* in adult acinar cells harboring oncogenic *Kras* mutation accelerated acinar to ductal reprogramming leading to mucinous PDAC precursor lesions in mice. ATAC-seq analysis showed reduced chromatin accessibility, and further studies pointed that these sites correlate with access of transcription factors to enhancers related to acinar identity genes [94]. These observations support the tumor-suppressive role of ARID1A in pancreas. 

#### 4.1.2. ARID1B 

*ARID1B* encodes an alternate DNA-binding subunit of the human SWI/SNF complex. The genomic alteration and mutation frequency of *ARID1B* is lower compared to *ARID1A* (Table 1). ARID1B expression is reduced in PDAC tumors (Table 2), and the gene is proposed to have a tumor-suppressive role. A limited number of studies in cell lines have been done to characterize the function of ARID1B. For instance, the pancreatic cancer cell line MIA PaCa-2 has a homozygous deletion of *ARID1B* and ectopic expression of ARID1B severely inhibited colony formation and anchorage independent growth of the cells [84]. Similarly, *ARID1B* knockdown promoted the growth-factor independent growth in normal human pancreatic duct epithelial (HPDE) cell line [20]. In addition, ARID1B transcription can also be epigenetically regulated through methylation [84]. 

ARID1A and ARID1B are mutually exclusive, and few studies have been done to characterize the functional dependency between ARID1A and ARID1B in cancer. *ARID1A*-deficient pancreatic cancer cells are selectively sensitive to *ARID1B* knockdown and have lower viability compared to ARID1A-expressing cells [21]. Similar findings were observed in a previous study which concluded that ARID1B is the preferential gene required for the survival of *ARID1A*-mutant cancer cell lines and loss of *ARID1B* in *ARID1A*-deficient background destabilized SWI/SNF and impaired proliferation, suggesting that ARID1B might be a potential target in *ARID1A*-mutant cancers [95]. 

#### 4.1.3. SMARCA2 

SMARCA2 is one of the mutually exclusive catalytic subunits of the SWI/SNF complex. It is generally accepted that loss of SMARCA2 expression is associated with formation of benign tumors [96]; however studies, of its role in PDAC mostly indicate an oncogenic function. Studies of patient samples have demonstrated a correlation between SMARCA2 expression, worse clinicopathological features, and worse survival [83,85,97] (Table 2). Limited mechanistic studies have been done to characterize the role of SMARCA2 in PDAC. In vivo studies using *SMARCA2*-silenced pancreatic cancer cells showed that mice had improved survival and decreased metastases [97]. Likewise, *SMARCA2* knockdown in cell lines resulted in decreased proliferation and reduced invasion [85,97]. Mechanistically, *SMARCA2* knockdown led to reduced activation of the JAK2/STAT3 pathway, inhibition of STAT3 phosphorylation and reduced transcription of STAT3 target genes [85]. Another study demonstrated the role of SMARCA2 in chemotherapy response. SMARCA2-downregulated pancreatic cancer cells had increased chemosensitivity to gemcitabine in vitro and in vivo [85]. Collectively, these studies suggest that further mechanistic studies are needed to delineate the role of SMARCA2 in PDAC. 

#### 4.1.4. SMARCA4 

SMARCA4 is the other mutually exclusive catalytic subunit of the SWI/SNF complex that has significant roles in pancreas development. Early embryonic pancreas-specific removal of *Smarca4* led to reduced multipotent pancreatic progenitor cell proliferation and resulted in pancreas hypoplasia [48], indicating its important role in modulating gene expression during development. *SMARCA4* is the second most frequently mutated gene of the SWI/SNF subunits in PDAC and is one of the well-studied SWI/SNF subunits. In most cases, SMARCA4 acts as a tumor suppressor; however, it has context-specific oncogene roles [88]. Several studies indicated that SMARCA4 expression is increased in pancreatic cancer tissues [83,85,86] (Table 2). Further studies demonstrated that loss of SMARCA4 in pancreatic and other tumors is associated with E-cadherin loss, vimentin upregulation, and EMT [98]. 

Interestingly, SMARCA4 has stage-specific roles during PDAC progression, as demonstrated by the studies done in IPMNs, which are precursor lesions of PDAC. Contrary to the PDAC samples, SMARCA4 expression is reduced or lost in IPMNs. Analysis of normal pancreatic epithelium by IHC showed strong expression of SMARCA4, whereas reduced expression or loss of SMARCA4 was observed in surgically resected IPMNs [87]. Other studies also confirmed the differential expression of SMARCA4 in IPMNs compared to PDACs. For example, SMARCA4 expression is higher in human PDAC samples compared to the IPMN lesions [88,89]. Further characterization studies utilizing *Kras*^G12D^ mouse models indicated the opposing roles of SMARCA4 in IPMN to PDAC progression. During early stages SMARCA4 acts as a tumor suppressor and inhibits dedifferentiation of ductal cells, whereas, at late stages, it induces EMT and promotes tumorigenesis [88]. Mechanistically, loss of *Smarca4* promoted dedifferentiation of pancreatic ductal cells expressing oncogenic Kras^G12D^ and led to development of IPMN lesions in vivo. Re-expressing SMARCA4 in a *Kras*^G12D^; *Smarca4*^f/^^f^ IPMN-derived cell line resulted in enhanced tumorigenicity and EMT characteristics [88]. Similarly, other studies showed that SMARCA4 acts as a tumor suppressor during the oncogenic *Kras*-induced IPMN-PDAC formation in vivo. Pancreatic loss of *Smarca4* and mutant *Kras* resulted in neoplastic cystic lesions that resembled human IPMNs and progressed to PDAC. Interestingly, opposing roles of SMARCA4 were detected during IPMN- and PanIN-PDAC progression, supporting the context-dependent and stage-specific roles of SMARCA4. Analysis of human samples revealed that reduction of SMARCA4 promoted PanIN-PDAC progression and resulted in poorer survival [89]. 

Several studies have been done to characterize the mechanistic role of SMARCA4. Characterization of SMARCA4-depleted IPMN-PDAC cells revealed the presence of repressive histone marks on the promoters of high-mobility group AT-hook 2 (*Hmga2*) gene, mediator of aggressive cancer phenotype, and other genes whose expression was reduced in IPMN-PDA [89]. Re-expressing SMARCA4 in a *Kras*^G12D^; *Smarca4*^f/f^ IPMN-derived PDAC cell line upregulated Hmga2 expression through binding to its promoter and activating its transcription [88,89]. In addition, SMARCA4 binding to *Sox9* regulatory elements was demonstrated [89]. Overexpression of Sox9 in *Kras*^G12D^; *Smarca4*^f/f^ pancreatic ductal cells blocked duct dedifferentiation and inhibited upregulation of progenitor markers [88]. 

Further studies demonstrated the role of SMARCA4 in cell proliferation and chemoresistance. *SMARCA4*-deficient or *SMARCA4*-depleted pancreatic epithelial cells demonstrated increased sensitivity to the DNA-damaging agents cisplatin, oxaliplatin, irinotecan, and 5-fluorouracil [58]. Likewise, SMARCA4 knockdown led to reversal of chemoresistance to gemcitabine in MIA PaCa-2 cells [86]. Gemcitabine resistance has been linked to Akt signaling, and SMARCA4 knockdown led to reduced activation of Akt and increased sensitivity of cells to gemcitabine [86]. Furthermore, knockdown of SMARCA4 in pancreatic cancer cell lines PANC-1 and MIA PaCa-2 led to reduced growth in vitro and in vivo [86]. Conflicting results regarding the role of SMARCA4 in cell proliferation were obtained in another study. Re-expression of SMARCA4 in *SMARCA4*-deficient pancreatic cancer cell lines PANC-1 and Hs700T led to senescence and reduced cell growth. It is possible that the conflicting results are due to differences in the expression levels of the SWI/SNF subunits among different pancreatic cancer cell lines. MIA PaCa-2 cells express SMARCA4, whereas SMARCA4 protein levels are undetected in PANC-1 [20,98,99,100] and Hs700T cells [20]. 

Similar to the ARID1A/ARID1B functional dependency, *SMARCA4* mutant cancer cells showed sensitivity to *SMARCA2* depletion [101,102]. Likewise, SMARCA2 dependency was observed in *SMARCA4*-deficient cancer cells [103]. In addition to a panel of *SMARCA2*-deficient tested cells, *SMARCA2*-deficient pancreas carcinoma HuP-T4 cells were dependent on SMARCA4 [103]. These studies indicate the presence of SMARCA2/SMARCA4 paralog dependency for the maintenance of ATPase activity of the SWI/SNF complex and represent a novel treatment strategy of targeting SMARCA2 in *SMARCA4*-mutant cancers and vice versa. 

#### 4.1.5. SMARCC1 

SMARCC1 is a core subunit of the SWI/SNF complex. Only one study has described the role of SMARCC1 in PDAC. Analysis of survival in recurrent PDAC pointed that SMARCC1 can be used as a predictor to gemcitabine therapy, as only SMARCC1-positive patients benefited from gemcitabine therapy [90]. Further studies in gemcitabine resistant clones of pancreatic cancer cell lines MIA PaCa-2 and PSN1 showed decreased expression of SMARCC1 [90]. IHC analysis demonstrated homogeneous nuclear staining of SMARCC1 in normal pancreatic ductal cells, whereas variable expression was observed in the pancreatic cancer lesions (Table 2). Mechanistically, SMARCC1 was identified as a tumor-suppressor gene in other cancer cell lines with roles in cell cycle and senescence [104]. SMARCC1 promoted breast cancer progression and metastasis through being recruited to unique chromatin regions, including the Myc target gene *GADD45a* [105]. Further studies are needed to characterize the role of SMARCC1 in PDAC. 

#### 4.1.6. ACTL6B 

*ACTL6B*, a paralog of *ACTL6A*, has not been studied extensively in cancer. ACTL6B is amplified in PDAC (3–24%, Table 1), and detailed understanding of its role in tumor progression is needed. The role of ACTL6B in neuronal development and differentiation has been analyzed; however, studies in cancer are missing. Neuronal development involves ACTL6A to ACTL6B switch of the SWI/SNF complex subunits. Loss of *ACTL6B* resulted in impaired dendritic growth [56,106]. Expression of ACTL6B in *ACTL6A*-deficent mouse embryonic stem cells rescued the cells from cell death and maintained their undifferentiated state, indicating that ACTL6A and ACTL6B might have redundant functions depending on the cell type [107]. Given the amplification frequency of ACTL6B observed in PDAC, further studies are needed to understand its role in tumorigenicity. 

Findings regarding the roles of the remaining subunits of the SWI/SNF subfamily complexes in PDAC are summarized in Table 3. 

### 4.2. ISWI Subfamily

#### 4.2.1. BPTF

BPTF is a component of the nucleosome remodeling factor (NURF) complex of the ISWI subfamily. BPTF expression has been shown to be increased in several cancer types and was associated with tumor progression and worse survival [147,148,149,150,151]. Although *BPTF* has been associated with deletions, amplifications, and mutations in PDAC (Table 1), functional studies on its role in PDAC are limited. Mechanistically, BPTF-activated human telomerase reverse-transcriptase (hTERT) expression and promoted stemness, proliferation, tumor growth, and metastasis associated with liver cancer [151]. Similarly, studies in other cell lines indicated that BPTF promoted proliferation and invasiveness in vitro [147,149,152]. Furthermore, other studies indicated that BPTF was associated with MYC signaling and promoted tumorigenesis [147,153]. In fibroblasts, *BPTF* knockdown led to changes in chromatin accessibility, reduced c-MYC recruitment to DNA, and decreased c-MYC-driven transcriptional signatures. BPTF knockdown suppressed the proliferation of pancreatic cancer cells and delayed the development of c-MYC-driven pancreatic tumors [153]. Taken together, these findings indicate that BPTF has an oncogenic role. 

In addition, BPTF expression was associated with chemoresistance. BPTF expression was associated with promoting resistance to BRAF inhibitors in melanoma [149], and its knockdown sensitized liver cancer cells to chemotherapeutic drugs [151]. 

Findings regarding the roles of the remaining subunits of the ISWI subfamily complexes in PDAC are summarized in Table 4. 

### 4.3. CHD Subfamily

#### 4.3.1. CHD1

CHD1 is a component of the CHD chromatin remodeling subfamily. CHD1 binds to histone marks associated with active transcription [186], maintains an open chromatin state, and promotes pluripotency in mouse embryonic stem cells [187]. Studies of CHD1 in PDAC are limited. A single study in pancreatic cancer cells suggested that CHD1 might have a pro-oncogenic function. In pancreatic cancer, the hPaf1 subunit of the human RNA polymerase II-associated factor (PAF) complex is overexpressed [188], and it interacts with and regulates the expression of CHD1 and the nuclear import of CHD1, facilitating the nucleosomal remodeling in pancreatic cancer cells [189]. The pro-oncogenic function of CHD1 is supported by studies in other cell lines. Studies in colorectal adenocarcinoma cells demonstrated that *KRAS* mutation is associated with elevated SUMOylation of CHD1 and other proteins that supported the anchorage independent growth of the cells [190]. Furthermore, in prostate cancer, CHD1 loss sensitized cells to DNA damage, caused DNA repair defects, and enhanced therapy response to DNA-damaging therapy and PARP inhibitors [191,192]. 

#### 4.3.2. CHD5 

CHD5 is a component of the CHD chromatin remodeling subfamily and is a tumor suppressor [61,193]. Upstream factors, including the WNT/β-catenin pathway, are involved in the transcriptional regulation of CHD5 [61,194]. Limited studies have been performed to assess the function of CHD5 in PDAC. IHC analysis showed that low CHD5 expression correlated with worse patient outcomes (Table 2) in PDAC [93], and similar results were observed in other cancers [195,196]. Epigenetic silencing of CHD5 through methylation has been observed in multiple cancer types [194,195,196,197]. Low CHD5 expression and CHD5 depletion in several pancreatic cancer cell lines has been associated with DDR activation [93]. Furthermore, CHD5 is a component of the NuRD transcriptional repressor complex [195]. CHD5 has been linked to WEE1, which is a key regulator of cell-cycle progression that can act as an oncogene [198]. CHD5 represses *WEE1* transcription in PANC-1 pancreatic cancer cells, thus acting as a tumor suppressor [199]. Similarly, WEE1 kinase inhibitor has recently shown promising results in combination therapy for PDAC [200]. Mechanistic studies in other cell types demonstrated that CHD5 expression suppressed expression of oncogenes, stem cell markers, and EMT markers in renal carcinoma cells [196]; and it resulted in reduced clonogenicity, cell proliferation, migration, and invasion in renal carcinoma cells and colorectal cancer cells [194,196]. 

#### 4.3.3. CHD7

CHD7 is a component of the CHD chromatin remodeling subfamily. Mutations in the *CHD7* gene cause a severe developmental human disorder, CHARGE syndrome [201], highlighting its role in neural stem cells and in development [202]. Mutations and/or altered gene expression of CHD7 are associated with various cancers, including breast cancer, gastric cancer, colon cancer [203,204,205], and PDAC (Table 1). CHD7 is also upregulated in gliomas, and mechanistic studies demonstrated that CHD7 overexpression enhanced cell migration and invasion in vitro and tumor growth in vivo [206]. Transcriptome analysis revealed that CHD7 altered the expression of adhesion molecules, stimulating cell motility and invasiveness [206]. 

A limited number of studies have focused on characterizing the role of CHD7 in PDAC. CHD7 is differentially methylated in PDAC [207]. CHD7 was dysregulated in over 90% of the analyzed PDAC samples, and low CHD7 expression was associated with increased survival in patients receiving adjuvant gemcitabine therapy [208]. Mechanistically, CHD7 depletion sensitized PDAC cells to gemcitabine by triggering DNA damage and delayed tumor xenograft growth [208]. CHD7 is amplified in PDAC (3.26–4.59%; Table 1), and further studies are needed to delineate its role. 

Findings regarding the roles of the ATPase subunits of the CHD subfamily complexes in PDAC are summarized in Table 5. 

### 4.4. INO80 Subfamily

#### 4.4.1. INO80

INO80 is the ATPase subunit of the INO80 complex. The majority of the studies focusing on the INO80 complex in cancer have been performed by using *INO80* knockdowns. Several studies have been done to characterize its oncogenic role in cancer and maintenance of stem cells; however, studies in PDAC are missing. INO80 is upregulated in cancer cell lines and human cancer tissues, including lung cancer, colon cancer, and melanoma [67,68,69,218]. Functional studies demonstrated that INO80 is required for proliferation, viability, clonogenicity, and anchorage-independent growth of cancer cells in vitro and tumor formation in vivo [67,68,218]. Supporting these findings, INO80 knockdown led to smaller tumors in vivo and downregulation of stem-cell-specific factors, reduced proliferation, and reduced migration in vitro [68,69,218]. Mechanistically, INO80 occupies enhancers near cancer-associated genes and promotes their expression, thus enhancing tumorigenicity [67,68]. 

Similarly, INO80 is involved in the renewal of embryonic stem cells (ESCs) by maintaining open chromatin architecture and selectively activating pluripotency genes [70]. Further studies pointed that INO80 might promote nucleosome depletion, as ATAC-seq studies of INO-80 silenced cells showed a significant increase in nucleosome occupancy at INO-80 bound regions [68], thus supporting its role in promoting an open chromatin state. 

#### 4.4.2. INO80C

INO80C is a core subunit of the INO80 complex that is involved in nucleosome recognition [219]. A recent study pointed to its role as a novel potential tumor suppressor in *KRAS*^MUT^ PDAC and colorectal cancer (CRC) xenograft tumors. Analysis of TCGA data revealed frequent deep deletions of *INO80C* in PDAC, and association between INO80C deletion and worse prognosis of patients with *KRAS*^MUT^ PDAC and CRC was observed. Knockdown of *INO80C* in *KRAS*^MUT^ PDAC and CRC cell lines demonstrated enhanced growth of the xenografts in vivo [220]. Given the high frequency of deletions of INO80C in PDAC samples (2.17–18.35%, Table 1), further studies are needed to characterize its role. 

Limited studies have been conducted in order to characterize the role of the other INO80 subfamily complexes in cancer [67,68,220]. Several reviews are focused on the roles of the INO80 subfamily complexes [51,219,221,222,223]. The remaining two complexes of the INO80 subfamily (Snf2-related CBP activator protein (SRCAP) and p400 [224,225] and their role in PDAC are not discussed in this review due to limited number of PDAC-specific studies. 

Findings regarding the roles of the remaining subunits of the INO80 complex in PDAC are summarized in Table 6. 

### 4.5. SWI/SNF and INO80 Subfamilies 

#### 4.5.1. ACTB

*ACTB* encodes β-actin, which is increased in PDAC and other cancers [267,268]. Studies in gastric cancer have indicated a higher level of β-actin in the primary tumor and a correlation between higher β-actin expression and lymph node metastasis [269]. Rearrangement of the actin cytoskeleton occurs during EMT [270] and, not surprisingly, downregulation of β-actin inhibited migration of gastric cancer cells [269]. As β-actin is implicated in cancer progression [267,271], further studies are needed to determine its role in PDAC. 

It is important to distinguish the roles of cytosolic and nuclear β-actin in tumorigenesis. The nuclear isoform of β-actin is part of several chromatin remodeling complexes (SWI/SNF and INO80 p400). Nuclear β-actin was involved in the quiescence of breast epithelial cells, as growth factor removal induced downmodulation of nuclear β-actin, which led to growth arrest [272]. Signals from the extracellular matrix (ECM) decreased nuclear-actin export, resulting in accumulation of nuclear actin and activation of growth-related transcription and malignant progression of breast cancer [273]. Nuclear actin could be a potential therapeutic target, as doxorubicin treatment resulted in nuclear actin aggregates and affected the recruitment of nuclear DNA-damage repair factors [274]. 

Interesting findings have linked mechanotransduction to actin dynamics and modulating β-actin localization. High mechanical stress (stretched cells) led to nuclear β-actin/F-actin localization at the whole nucleoplasm compared to a perilaminar distribution of nuclear β-actin/F-actin in low-mechanical-stress cells. β-actin polymerizes to form filamentous (F) actin, which is an important component of the cytoskeleton and plays a role in motility [275]. These findings highlight the role of nuclear actins in linking extracellular mechanical signals to chromatin regulation. 

ACTB is mostly regarded as a housekeeping gene and is widely used as an endogenous reference for quantification of protein/gene expression studies. Its differential increase in cancer samples suggests that it might not be an appropriate endogenous control. The comparison of four pancreatic ductal cell lines demonstrated that β-actin protein levels did not vary significantly across the cell lines. However, analysis of RNA seq data of 41 PDAC cell lines demonstrated that *ACTB* is one of the genes with the highest standard deviations [276,277]. Other studies have also demonstrated that β-actin might not be an appropriate control for real-time quantitative reverse-transcription PCR [278,279,280]. 

#### 4.5.2. ACTL6A

ACTL6A is a component of the SWI/SNF complex and INO80 subfamily INO80 and P400 complexes. It encodes actin related proteins (ARPs) that resemble actin and have roles in chromatin modification and histone acetylation. Amplifications of *ACTL6A* are associated with PDAC (Table 1); however, mechanistic studies in PDAC are missing. Several studies demonstrated that *ACTL6A* is amplified and upregulated in different cancers [137,281,282,283,284,285]. ACTL6A has a protumorigenic function and its expression level correlated with worse clinicopathological features in liver cancer and in colon cancer [283,284]. Mechanistically, ACTL6A overexpression promoted migration and invasion and induced EMT in vitro [283,284] and promoted tumor growth and metastasis in a mouse liver cancer xenograft model [283]. Further studies demonstrated that ACTL6A targets SOX2 expression, which activates Notch1 signaling, leading to EMT [282,283]. 

Moreover, ACTL6A is associated with stem cell maintenance [107,281,282,286], including activation of the Hippo-YAP pathway [281], Nanog binding to pluripotency genes, and repression of differentiation genes [286], which might explain its role in cancer. As ACTL6A promotes a stem-cell-like state, it is not surprising that its levels are increased in cancer. Few studies have been done to delineate the chromatin-specific role of ACTL6A. ACTL6A binds to core histones and might modulate the interaction of the chromatin-modifying complexes with nucleosomes [287,288]. *ACTL6A* depletion accelerated the degradation of SMARCA4 and SMARCC2 and destabilized SMARCA4 chromatin remodeling complexes in several human cell lines [288]. Moreover, majority of the endogenous ACTL6A proteins are associated with SMARCA4 in the nucleus; thus, they are involved in chromatin modification [288]. 

## 5. Therapeutic Targeting of Chromatin Remodeling in Pancreatic Cancer

Chromatin remodeling complexes constitute only a portion of the epigenetic regulation mechanisms, and their roles in tumorigenesis have been highlighted in multiple studies. Genomic lesions are highly prevalent in ATP-dependent chromatin remodelers; however, specific small molecules that effectively target these complexes are limited. The complexity of the ATP-dependent chromatin remodelers poses a significant challenge for their pharmacological targeting. Several different approaches that involve siRNA libraries, inhibitor libraries, and computational modeling have been utilized to identify novel molecules. The majority of the studies have been focused on inhibitors that target the bromodomain domain or the ATPase domain of the subunits. Currently, there are very few potent and selective molecules targeting the subunits of the ATP-dependent chromatin remodeling complexes. 

BPTF expression was associated with c-MYC signaling and tumorigenicity in multiple studies. A recent computational docking-based virtual screening identified C620-0696 as a potential inhibitor of BPTF. The addition of C620-0696 to BPTF overexpressing lung cancer cells resulted in cytotoxicity, suppression of c-Myc expression, and inhibition of migration and colony formation, indicating that targeting of BPTF can be further explored as a treatment strategy [289]. Another recent screening study demonstrated that GSK2801, an inhibitor of BAZ2A/B bromodomains of the ISWI complexes and BRD9 of the SWI/SNF complex, synergizes with bromodomain and extra-terminal motif (BET) inhibitors to induce apoptosis in triple-negative breast cancer in vitro [290]. GSK2801 did not result in a significant growth inhibition as a single agent, indicating the need for combinatorial treatment screens. 

PFI-3 is a small molecule inhibitor that selectively targets the bromodomain domain of family VIII bromodomain proteins, which include SMARCA2, SMARCA4, and PBRM1 subunits of SWI/SNF complexes. Two studies showed that PFI-3 could not inhibit proliferation [291,292]. Further characterization studies demonstrated that PFI-3 cannot displace endogenous, full-length SMARCA2 from the chromatin, which raises the possibility that it cannot disrupt SMARCA2/SMARCA4-chromatin interaction. Further in vitro studies demonstrated that targeting the ATPase activity of SMARCA2 and SMARCA4 might be a more potent target in cancer [291,292,293]. 

Promising results have been observed with the active DNA-dependent ATPase A Domain inhibitor (ADAADi), which is the first-in-class inhibitor that inhibits the catalytic ATPase domain of the SWI2/SNF2 family members. ADAADi’s are natural products of aminoglycoside-resistant bacteria that compete with respect to the DNA effector needed for ATP hydrolysis of ATPases. Biochemical studies demonstrated that a subset of the ADAADi’s disrupted ATP-dependent nucleosome activity [294]. Studies in triple-negative breast cancer cell lines demonstrated that ADAADi’s decreased cell proliferation. However, it only targeted a subset of cells preferentially as treatment of cells with reduced SMARCA4 expression did not respond to the treatment [292]. Moreover, ADAADi treatment blocked drug efflux transporter gene expression; thus, it sensitized cells to chemotherapeutic drugs [292]. Studies in other cells lines demonstrated that ADAADi disrupted EMT, inhibited cell migration, and induced apoptosis. Treatment with ADAADi led to transcriptional changes which included repression of the tumor-promoting genes and upregulation of the pro-apoptotic and tumor-suppressors genes [293]. 

Recent screening study utilizing the proteolysis targeting chimera (PROTAC) technology has identified degraders of the SWI/SNF complex ATPase subunits SMARCA2/SMARCA4 and DNA binding subunit PBRM1 [295]. PROTACs degrade target proteins through recruitment of the ubiquitin proteasome system, which is achieved by using a target-binding ligand linked to a E3 ligase–binding ligand. In this case, the PROTAC ligand was targeted against the bromodomain motif of the proteins. The optimized PROTAC chemical probe ACBI1 resulted in complete degradation of SMARCA2/SMARCA4 and PBRM1. ACBI1 inhibited cell proliferation and induced apoptosis in leukemia cell lines with an intact BAF complex and SMARCA4-mutant cancer cells. These findings suggest that targeted degradation of BAF complex ATPases can be used as a potential treatment strategy. 

Taken together, these studies suggest that targeting different domains of the ATPase subunits of the chromatin remodeling complexes can be used as a potential cancer treatment strategy. However, further studies are needed to determine their specificity and effect in normal cells and cancer cells. Multiple challenges are associated with identifying specific small compounds or probes against the subunits of the ATP-dependent chromatin modifying complexes. The compounds/probes have to be specific and target the critical domain of the subunits. In addition, as demonstrated by the PFI-3 study, targeting of the correct domain might not influence the activity of the complex within the cells. In addition, combinatorial treatment screening assays might need to be implemented to test the synergistic effect of drugs, as demonstrated by the GSK2801/BET inhibitor screening study. 

## 6. Conclusions

ATP-dependent chromatin remodeling complexes are involved in the dynamic regulation of gene transcription. Perturbation of the ATP-dependent chromatin remodeling complexes has been associated with cancer, including PDAC. Although the expression of these genes appears to have an impact on PDAC progression and chemoresistance, functional data regarding the role of majority of the individual subunits in PDAC is missing. Detailed understanding of the effect of chromosomal aberrations and mutations associated with components of the ATP-dependent chromatin remodeling complexes in oncogenesis might lead to the discovery of downstream therapeutic targets. 

Currently, the ATP-dependent chromatin remodeling complexes are divided into four subfamilies. We noticed that the mechanistic studies have focused on a limited number of complexes, particularly the SWI/SNF subfamily complexes. The remaining subfamilies (ISWI, CHD, and INO80) have not been studied extensively in PDAC, and detailed studies to understand their involvement in PDAC are urgently needed. A noncanonical BAF complex and several subunits of the SWI/SNF complexes (BCL7, BRD7, and BRD9) have been recently identified and detailed studies regarding their function are missing. Multiple subunits, including *ACTL6B, SMARCD3, DPF1, DPF2, BCL7B, BCL7C, BRD9, BICRA, BICRAL, SS18, SS18L, CHRAC1, INO80C, RUVBL2, UCHL5,* and *TFPT*, display a high percentage of chromosomal aberrations and/or mutations in PDAC; therefore, mechanistic studies are needed to delineate their role in transcriptional regulation and oncogenesis. 

Expression of several of the subunits (*SMARCA4, BCL11B, BPTF, SMARCA2, CHD1, CHD4, CHD7, SMARCD1,* and *SMARCE1*) also correlated with chemoresistance and chemosensitivity. Therefore, further mechanistic understanding of their function might be important to identify pathways that can increase sensitivity to current drug regimens. 

Targeting the ATP-dependent chromatin remodeling complexes has demonstrated promising results in decreasing cancer cell proliferation in vitro. Recent studies, using either ADAADi’s or PROTACs, have focused on targeting the bromodomain domains and the ATPase domains of the SWI/SNF complex ATPase subunits SMARCA2 and SMARCA4. Another study identified a bromodomain inhibitor, GSK2801, that targets BAZ2A/B and BRD9 and has shown successful results in a combinatorial treatment. It would be beneficial to test these compounds in pancreatic cancer cell lines. 

In conclusion, ATP-dependent chromatin remodeling complexes modulate gene expression, and, with few exceptions, detailed studies regarding their role in PDAC are lacking. Studies exploring their mechanistic roles in PDAC are needed for our understanding of PDAC chromatin biology, identification of novel therapeutic targets, and development of specific cancer therapeutics. Furthermore, the expression of individual subunits or complexes can be used as prognostic markers to predict response to therapy. 

## Figures and Tables

**Table 1:** Chromosomal copy number alteration (CNA) frequency (%) and mutational frequency (%) of genes encoding subunits of the ATP-dependent chromatin remodeling complexes.

Gene		Deep Deletions		Amplifications		Fusions	Somatic Mutations			
		UTSW	TCGA	UTSW	TCGA	TCGA	UTSW	QCMG	TCGA	ICGC
*SWI/SNF subfamily*	ARID1A	8.26	1.09			0.54	6.42	7.57	4.89	4.04
	ARID1B	3.67	0.54					0.52	1.09	
	PBRM1	4.59	0.54	1.83			0.92	1.83	2.72	1.01
	SMARCA2	7.34	0.54	0.92	0.54	0.54		0.78	0.54	1.01
	SMARCA4	0.92	1.09	5.55			1.84	2.87	1.09	1.01
	SMARCB1			2.75			0.92	0.52	0.54	
	SMARCC1	4.59	0.54	3.67	0.54				0.54	
	SMARCC2	1.83		4.59	1.63			0.26	0.54	
	ACTB *			15.60	0.54		2.75		0.54	
	ACTL6A *			2.75	2.17					
	ACTL6B	0.92		23.85	3.26					
	SMARCD1	1.83	0.54					0.52	0.54	
	SMARCD2			3.67	1.63				0.54	
	SMARCD3			11.01	0.54				0.54	
	SMARCE1	0.92		4.59	1.63			0.26	1.09	1.01
	PHF10	3.67	0.54				0.92	0.26	0.54	
	DPF1			16.51	4.89				0.54	
	DPF2			9.17					0.54	
	DPF3	1.83		0.92				1.04	0.54	
	ARID2	5.5	0.54	0.92			1.83	2.09	1.63	3.03
	BRD7	2.75		1.83	0.54			0.26		
	BCL7A	1.83				0.54				
	BCL7B			11.93	1.09			0.26		
	BCL7C	0.92		6.42				0.26		
	BCL11A	1.83		1.83			0.92	0.78	0.54	1.01
	BCL11B	2.75		0.92				1.04	1.09	
	BRD9			13.76	0.54		1.83	0.26		
	BICRA	0.92		12.84	1.09		0.92	0.52	0.54	
	BICRAL	1.83		6.42	1.63			0.52	1.09	
	SS18	10.09	0.54	5.5	4.35	0.54	0.92	0.26	0.54	
	SS18L1	0.92		11.01	1.09					
*ISWI subfamily*	SMARCA1				0.54		1.83	0.78	1.09	
	SMARCA5	5.5					0.92	0.26		
	BAZ1B			6.42	1.09		0.92	0.26	0.54	
	BAZ2A	1.83		3.67	1.63			0.26	0.54	
	RSF1			0.92			0.92	0.26		
	BAZ1A	1.83		2.75			0.92	0.26		
	CHRAC1	0.92	0.54	12.84	8.7	0.54			0.54	
	POLE3	0.92	0.54	1.83						
	BPTF	3.67		2.75	2.17		0.92	0.52	2.72	
	CECR2	0.92						0.78	1.09	
	RBBP4	5.5		0.92						
	RBBP7		0.54		0.54			0.78	0.54	1.01
*CHD subfamily*	CHD1	3.67		1.83			0.92	0.78		
	CHD2			5.5	2.17		1.83	1.04	0.54	
	CHD3	5.5	0.54	0.92				0.26	1.09	
	CHD4	0.92		7.34	2.72		1.83	0.52	1.09	
	CHD5	7.34	1.09	5.5	1.63			0.78	1.09	
	CHD6	0.92		4.59	0.54			1.57	2.17	
	CHD7	0.92		4.59	3.26		0.92	0.78	0.54	2.02
	CHD8	0.92			0.54			0.78	1.09	1.01
	CHD9	1.83		2.75	0.54		1.83	0.52	1.09	
*INO80 subfamily*	INO80			4.59	0.54			1.31	0.54	1.01
	ACTR5	0.92		3.67				0.52	0.54	
	ACTR8	5.5		0.92				0.26	0.54	
	INO80B	0.92		4.59				0.26		
	INO80C	18.35	2.17	0.92	1.63			0.26	0.54	
	INO80D	0.92		1.83	1.63			0.26		
	INO80E			7.34				0.26	0.54	
	RUVBL1			2.75	1.09					
	RUVBL2	2.75		12.84	1.09			0.26	0.54	
	YY1	3.67		4.59				0.26	0.54	
	MCRS1	2.75	0.54	2.75				0.26		
	NFRKB	0.92		2.75	0.54	0.54	0.92	0.52	0.54	1.01
	UCHL5	2.75	0.54	10.09	2.17			0.52		
	TFPT	0.92		11.93	1.09					

Note: Frequencies are based on sequencing data analyzed through cBioPortal [73,74]. Percentages were calculated using data derived from the following studies: UTSW (109 samples, CNA and exome sequencing) [21], TCGA PanCan (184 samples, CNA and exome sequencing) [75,76,77,78,79,80], QCMG (383 samples, exome sequencing) [81], ICGC (99 samples, exome sequencing) [22] (percentages were color coded: blue-deep deletions, red-amplifications, purple-fusions, green-somatic non-silent mutations). Germline mutations were not detected for any of the listed genes. Only ATPase subunits are included as part of the CHD subfamily complexes. SWI/SNF noncanonical complex ncBAF and INO80 subfamily complexes p400 and SRCAP are not included in the analysis. * ACTB and ACTL6A are also components of the INO80 subfamily complexes.

**Table 2:** Summary of immunohistochemistry (IHC) analysis for subunits of ATP-dependent chromatin remodeling complexes in PDAC patient samples.

Protein	Summary of Findings
ARID1A	Expression absent in 22% of surgically resected IPMN and in 36% of PDAC samples [82]. ARID1A expression was absent or low in 61% of the gastric and 10% of pancreaticobiliary IPMN subtypes [50]. Deficiency was significantly associated with poor outcome in PDAC [21]. Another study concluded that there was no association between ARID1A expression and clinicopathological features or overall survival [83].
ARID1B	Reduced/nondetectable expression in pancreatic tumor compared to matched normal samples. Reduction in expression was more noticeable in advanced-stage tumors [84].
PBRM1	High PBRM1 expression was related to smaller pancreatic tumor size. PBRM1^high^ patients had improved 5-year survival rate compared to PBRM1^low^ patients [83].
SMARCA2	SMARCA2 expression was associated with worse clinicopathological features in pancreatic cancer cases. The survival rate of SMARCA2^high^ patients was significantly worse compared to SMARCA2^low^ patients [83]. SMARCA2 expression correlated significantly with tumor histological grade. SMARCA2^high^ group (56.5%) had significantly worse survival rate compared to the SMARCA2^low^ (43.5%) group [85].
SMARCA4	SMARCA4 expression was increased in pancreatic cancer tissues [83,86]. Association between SMARCA4 expression, histology, and stage was observed: SMARCA4^high^ correlated with stage IV disease [83]. SMARCA4 has been shown to be expressed heterogeneously in pancreatic cancer tissues. Trend between SMARCA4 expression and tumor grade was observed, and SMARCA4^low^ group had a tendency for higher survival rate [86]. SMARCA4 expression was lost in 8.3% and reduced in 53.3% of the IPMN cases, and decreased SMARCA4 expression correlated with increased dysplasia in IPMN lesions. High-grade IPMNs had more frequent loss (76%) compared to intermediate-grade (52%) and low-grade IPMNs (28%) [87]. SMARCA4 expression was higher in PDAC compared with its precursor IPMN lesions [88,89].
SMARCC1	Nuclear staining of SMARCC1 was detected in normal pancreatic ductal cells, whereas variable expression was observed in pancreatic cancer lesions (47% had positive staining and 53% had negative staining). SMARCC1 expression did not correlate with patient survival [90].
BCL7B	BCL7B was overexpressed in pancreatic cancer. BCL7B^high^ was associated with shorter survival time. Normal pancreatic ducts did not stain for BCL7B [91].
UCHL5	Both nuclear and cytoplasmic localization was observed in human PDAC tissues and positive nuclear UCHL5 expression was associated with better prognosis in PDAC patients [92].
CHD5	CHD5 expression correlated with patient survival. Low CHD5 expression predicted worse survival in patients with resected PDAC receiving adjuvant chemotherapy [93].

**Table 3:** Functional studies of subunits of the SWI/SNF complexes in PDAC (or other cancers).

SWI/SNF Subfamily	
Subunit	Protein Name/Functional Studies
ARID1A (BAF250A)	AT-Rich Interaction Domain 1A. Most mutated subunit in pancreatic cancer. Tumor suppressor. See Section 4.1.1.
ARID1B (BAF250B)	AT-Rich Interaction Domain 1B. Tumor suppressor. See Section 4.1.2.
PBRM1 (BAF180) *	Polybromo 1. Tumor suppressor. High incidence of truncating mutations [108] and association between PBRM1 loss and tumor response to immunotherapy in clear cell renal carcinoma [109]. PBRM1-deficient renal carcinoma tumors have a distinct transcriptional signature linked to hypoxia and other altered signaling pathways [109,110]. PBRM1 has been shown to regulate stress response in normal epithelial cells and its deletion led to increased proliferation and EMT [111].
SMARCA2 (BRM, BAF190B)	SWI/SNF Related, Matrix Associated, Actin Dependent Regulator of Chromatin, Subfamily A, Member 2. Tumor-suppressive role. See Section 4.1.3.
SMARCA4 (BRG1, BAF190A)	SWI/SNF Related, Matrix Associated, Actin Dependent Regulator of Chromatin, Subfamily A, Member 4. Tumor-suppressor and oncogenic roles depending on stage of tumor progression. See Section 4.1.4.
SMARCB1 (BAF47, INI1, hSNF5) *	SWI/SNF Related, Matrix Associated, Actin Dependent Regulator of Chromatin, Subfamily B, Member 1. Tumor suppressor linked to pathways associated with tumor proliferation and progression [112].
SMARCC1 (BAF155)	SWI/SNF Related, Matrix Associated, Actin-Dependent Regulator of Chromatin Subfamily C Member 1. See Section 4.1.5.
SMARCC2 (BAF170) *	SWI/SNF Related, Matrix Associated, Actin-Dependent Regulator of Chromatin Subfamily C Member 2. Frameshift mutations in *SMARCC2* in gastric and colorectal cancers with microsatellite instability [113].
ACTB	Actin Beta. See Section 4.5.1.
ACTL6A (BAF53A)	Actin Like 6A. See Section 4.5.2.
ACTL6B (BAF53B) *	Actin Like 6B. Aberrant promoter methylation observed in esophageal cancer, liver cancer, and prostate cancer [114,115,116]. See Section 4.1.6.
SMARCD1 (BAF60A) *	SWI/SNF Related, Matrix Associated, Actin-Dependent Regulator of Chromatin, Subfamily D, Member 1. Interacts with p53 and mostly acts as a tumor suppressor [117,118]. Decreased expression in ovarian cancer [119] and in lung cancer [117]. SMARCD1 sensitized lung cancer cells to cisplatin-induced apoptosis [117], and its reduced expression triggered cellular senescence in hepatocytes [120]. Opposite results in gastric cancer: overexpressed in gastric cancer tissues and correlated with worse survival outcomes [121].
SMARCD2 (BAF60B) *	SWI/SNF Related, Matrix Associated, Actin Dependent Regulator of Chromatin, Subfamily D, Member 2. Highly expressed in pancreas [122], potential tumor suppressor in leukemia [123]. Involved in chromatin opening of hepatic genes and lineage conversion [124].
SMARCD3 (BAF60C) *	SWI/SNF Related, Matrix Associated, Actin Dependent Regulator of Chromatin, Subfamily D, Member 3. Induced Wnt5a signaling and controlled EMT in breast cancer [125]. Amplified in PDAC (Table 1).
SMARCE1 (BAF57) *	SWI/SNF Related, Matrix Associated, Actin Dependent Regulator of Chromatin, Subfamily E, Member 1. Promoted invasive and metastatic progression of breast cancer through upregulation of proteases that degrade ECM by forming a SWI/SNF-independent complex [126]. High expression in metastatic prostate cancer [127]. SMARCE1 loss induced EGFR expression, activated AKT and ERK signaling in lung cancer and conferred resistance to MET and ALK inhibitors [128]. Knockdown led to decreased cell growth and increased sensitivity to anticancer agents in ovarian cancer and breast cancer cell lines [129].
PHF10 (BAF45A) *	PHD Finger Protein 10. Might be neuron specific [56], required for cell proliferation in normal fibroblasts [130]. Tumor suppressor role in uveal melanoma [131].
DPF1/3/2 BAF45B/C/D) *	Double PHD Fingers 1/3/2. Rarely mutated in cancers [132].
ARID2 (BAF200) *	AT-Rich Interaction Domain 2. Tumor suppressor in hepatocellular carcinoma [133,134].
BRD7 *	Bromodomain-Containing Protein 7. Tumor suppressor involved in tumor development and progression in multiple cancers [135].
BRD9, BICRA (GLTSCR1), BICRAL (GLTSCR1L) *	Bromodomain Containing 9/ BRD4 Interacting Chromatin Remodeling Complex Associated Protein/ BRD4 Interacting Chromatin Remodeling Complex Associated Protein Like. Components of a newly identified noncanonical SWI/SNF complex involved in maintaining pluripotency in mouse embryonic stem cells [136]. Amplified in several cancers and may act as oncogenic drivers [137,138].
BCL7 (A/B/C) *	BAF Chromatin Remodeling Complex Subunit BCL7A/B/C. Accumulated in the cell protrusions of migrating pancreatic cells, involved in motility and invasiveness through CREB signaling pathway [91]. Tumor suppressor negatively regulating the Wnt-signaling pathway in gastric cancer cells [139].
BCL11 (A/B) *	BAF Chromatin Remodeling Complex Subunit BCL11A/B. BCL11A: Highly expressed in breast cancer and lung cancer, involved in cancer stemness and tumorigenesis [140,141,142]. BCL11B: overexpression led to chemoresistance in T-cell lines [143], acted as a tumor suppressor in T-cell acute lymphoblastic leukemia [144]. Downregulation in intestinal crypt cells increased expression of β-catenin genes, promoting tumor development [145].
SS18/SS18L1 (CREST) *	SS18 Subunit of BAF Chromatin Remodeling Complex/ SS18L1 Subunit of BAF Chromatin Remodeling Complex. Involved in neural development, and links Ca^2+^ signaling and chromatin reorganization [146].

Note: * No/limited mechanistic studies in PDAC.

**Table 4:** Functional studies of subunits of the ISWI complexes in PDAC (or other cancers).

	ISWI Subfamily
Subunit	Protein Name/Functional Studies
SMARCA1 (SNF2L) *	SWI/SNF Related, Matrix Associated, Actin-Dependent Regulator of Chromatin, Subfamily A, Member 1. Expression was decreased in malignant melanoma; depletion in HeLa cells led to activated Wnt signaling, increased proliferation and migration [154]. Expression was not detected in normal pancreas [154]. SMARCA1 depletion in cancer cells led to increased apoptosis, DNA damage response and upregulation of genes related to cell-cycle checkpoint arrest [155].
SMARCA5 SNF2H) *	SWI/SNF Related, Matrix Associated, Actin-Dependent Regulator of Chromatin, Subfamily A, Member 5. Expressed in human pancreas [154]. Increased in gastric cancer [156], breast cancer [157], and liver cancer [158]. Activated Wnt/β-catenin signaling [158] and promoted cancer cell proliferation, colony formation and invasion [157,158]. Depletion in HeLa cells led to apoptotic phenotype [154]. Interacts with CCCTC-binding factor (CTCF) and is associated with chromatin to regulate transcription [159]. Involved in DNA repair [160,161]. Required for proliferation and differentiation of hematopoietic stem cells [162].
BAZ1B (WSTF) *	Bromodomain Adjacent to Zinc Finger Domain 1B. Knockdown decreased melanoma tumor growth [163]. In lung cancer models, overexpression promoted proliferation and invasion through activating the PI3K/Akt and IL-6/STAT3 signaling pathways [164]. Involved in DNA damage response [165]. Promoted cell growth and reduced DNA-damage induced cell death in HeLa cells [59].
BAZ2A (TIP5) *	Bromodomain Adjacent to Zinc Finger Domain 2A. Upregulated in the serum of pancreatic cancer patients. Interacts with p53 and is involved in histone acetylation [166]. Overexpressed in prostate cancer and contributed to cell proliferation and viability. Associated with the CIMP molecular subtype and interacted with EZH2 to coordinate epigenetic silencing in prostate cancer cells [167]. It is also a part of the nucleolar remodeling complex (NoRC): involved in heterochromatin formation at telomeres and centromeres, thus maintaining genome stability [166,168].
RSF1 *	Remodeling and Spacing Factor 1. Overexpressed in ovarian cancer and other cancers [169,170,171]. Overexpression has been associated with poor prognosis in ovarian cancer patients. It is a co-activator of NF-kB signaling [172] and is involved in the development of chemoresistance in ovarian cancer cells [172,173]. Identified as a potential oncogene in breast cancer, overexpression led to increase the colony formation ability in vitro and enhanced tumorigenesis and invasion in vivo [174]. Interacts with cyclin E1 and promotes tumorigenesis [169]. Increased RSF1 expression induced chromosomal instability [170]. Involved in DDR and DNA repair [175].
BAZ1A (ACF1) *	Bromodomain Adjacent to Zinc Finger Domain 1A. Promoted cell growth after DNA damage and reduced DNA-damage induced cell death in HeLa cells [59]. Knockdown induced senescence associated phenotype through upregulation of SMAD3 [176].
BAZ2B *	Bromodomain Adjacent to Zinc Finger Domain 2B. Newly added to the ISWI complexes [59]. Paralogue of BAZ2A. Histone binding protein [177].
CHRAC1 (CHRAC15) *	Chromatin Accessibility Complex Subunit 1. Identified as a driver gene in breast cancer regulating proliferation [178]. Amplified in PDAC.
POLE3 (CHRAC17) *	DNA Polymerase Epsilon 3. Involved in chromatin remodeling and DNA replication [179], regulated by MYC [180,181]. Polysomes of SMAD4-/- BxPC3 cells had increased level of POLE3, thus it might contribute to the genomic instability in PDAC [181]. POLE3 proofreading mutations in endometrial cancer have been associated with higher T cell content and antitumor response [182].
BPTF *	Bromodomain PHD Finger Transcription Factor. Protumorigenic role. See Section 4.2.1.
CECR2 *	CECR2 Histone Acetyl-Lysine Reader. Histone acetylation modulator protein [183]. Identified as a DNA damage response protein [184], involved in neurulation [185].

Note: * No/limited mechanistic studies in pancreatic cancer.

**Table 5:** Functional studies of the ATPase subunits of the CHD complexes in PDAC (or other cancers).

CHD Subfamily	
Subunit	Protein Name/Functional Studies
CHD1 *	Chromodomain Helicase DNA Binding Protein 1. See Section 4.3.1.
CHD2 *	Chromodomain Helicase DNA Binding Protein 2. Tumor suppressor role in chronic lymphocytic leukemia [209]. Hypomethylated in PDAC [207]. Required to maintain the differentiation potential of mouse ESCs [210].
CHD6 *	Chromodomain Helicase DNA Binding Protein 6. A cancer driver and key regulator of the oxidative DNA damage response [211].
CHD7 *	Chromodomain Helicase DNA Binding Protein 7. See Section 4.3.3.
CHD8 *	Chromodomain Helicase DNA Binding Protein 8. Differentially methylated in PDAC [207]. Decreased expression in gastric cancer samples [212]. Negative regulator of the Wnt/β-catenin pathway [212,213], CHD8 knockdown in gastric cancer cells promoted proliferation [212].
CHD9 *	Chromodomain Helicase DNA Binding Protein 9. Decreased expression in CRC patient samples that correlated with worse prognosis [214].
CHD3 *	Chromodomain Helicase DNA Binding Protein 3. Component of the NuRD complex. Aberrant methylation was detected in advanced CRC and gastric cancer [215,216]. Overexpressed in cancers, including PDAC [216].
CHD4 *	Chromodomain Helicase DNA Binding Protein 4. Component of the NuRD complex. High expression was associated with tumor status, metastasis and poor prognosis in rectal cancer [217]. In CRC, CHD4 interacted with oxidative DNA damage sites and double-strand breaks recruiting repressive chromatin proteins that maintained epigenetic silencing of tumor suppressor genes [64]; high levels of CHD4 were associated with poor prognosis [64]. CHD4 was identified as a potential therapeutic target in CRC [63,64] as knockdown of CHD4 sensitized cells to DAC-induced cell death and reactivated tumor suppressor genes [63].
CHD5	Chromodomain Helicase DNA Binding Protein 5. Component of the NuRD complex. Tumor suppressor. See Section 4.3.2.

Note: * No/limited mechanistic studies in PDAC. Only the ATPase components are listed in the table. CHD members form multisubunit complexes [186], which are not discussed in this review.

**Table 6:** Functional studies of subunits of the INO80 complex in PDAC (or other cancers).

INO80 Subfamily (INO 80 Complex)	
Subunit	Protein Name/Functional Studies
INO80	INO80 Complex ATPase Subunit. See Section 4.4.1.
ACTL6A	Actin Like 6A. See Section 4.5.2.
ACTR5 (INO80M) *	Actin Related Protein 5. Increased in CRC [226], decreased in pancreatic tumors [227]. ACTR5 facilitates binding of INO80 complex to DNA, INO80 complexes lacking ACTR5 have reduced ATPase and chromatin remodeling activities in vitro [228]. Involved in nucleosome recognition [219].
ACTR8 (INO80N) *	Actin Related Protein 8. ACTR8 facilitates binding of INO80 complex to DNA, INO80 complexes lacking ACTR8 have reduced ATPase and chromatin remodeling activities in vitro [228].
INO80B *	INO80 Complex Subunit B. Regulates INO80 ATPase activity in vitro [219,229].
INO80C *	INO80 Complex Subunit C. Tumor suppressor role. See Section 4.4.2.
RUVBL1 (RVB1, Tip49a, pontin)/RUVBL2 (RVB2, Tip49b, reptin) *	RuvB Like AAA ATPase 1/2. RUVBL1: Required for efficient mitosis and proliferation of cells [230]. Expression is increased in HCC, CRC and other cancers, involved in cell invasion and EMT. Interacts with oncogene c-MYC and β-catenin. Roles in cell growth and viability [231,232,233,234,235,236,237,238]. In a mouse model of liver cancer, accumulation of E2f1 recruits the RUVBL1/RUVBL2 complex that opens the chromatin conformation at E2f target genes and amplifies the E2f transcriptional response during cancer progression. Can function as a separate complex, not involved in INO80 subfamilies [231]. Cytoplasmic RUVBL1 interacts with actin filaments at cell protrusions and thus promotes invasiveness and migration of PDAC cells [239], which is a role independent of its chromatin remodeling [240]. No other data in PDAC. RUVBL2: Expression is increased in HCC, CRC. Interacts with oncogene c-MYC and β-catenin. Roles in cell growth and viability [231,232,233,235,240,241,242,243]. Interacts with mutant p53 [244].
YY1	YY1 Transcription Factor. A zinc finger transcription factor, that can either repress or activate gene transcription by recruiting different cofactors. YY1 expression is increased in PDAC [245,246], higher YY1 levels are associated with oncogenic KRAS^G12D^ status in pancreatic cancer cell lines and patient samples [245]. YY1 regulates the expression of Snail1 and VEGF, promoting EMT and angiogenesis [247,248]. Conflicting results reporting its role as a tumor suppressor in inhibiting the migration, invasiveness and proliferation in PDAC cells [249,250,251]. Other studies also report a dual tumor suppressor and oncogenic role [247,252,253,254,255].
MCRS1 (MSP58) *	Microspherule Protein 1. Promoted proliferation, invasion and metastasis of lung cancer cells [256,257] and proliferation and tumor growth of colon carcinoma cells [258]. Increased in CRC [258,259,260]. Involved in mTORC1 activation, thus having an oncogenic role [259].
NFRKB	Nuclear Factor Related to KappaB Binding Protein. NFRKB binds to UCH37, disrupting the active site for ubiquitin binding and inhibiting its function [261].
UCHL5 (UCH37) *	Ubiquitin C-Terminal Hydrolase L5. UCHL5 deubiquitylase-dual roles component of INO80 and 26S proteasome [261]. Implicated in cancer [262,263]. Promotes Hedgehog signaling and TGFb-1 signaling [264,265].
TFPT *	TCF3 Fusion Partner. Translocations are involved in B-cell precursor acute lymphoblastic leukemia [266]

Note: * No/limited mechanistic studies in pancreatic cancer.
